# Optimized nitrogen fertilizer management enhances soybean (*Glycine max* (L.) Merril.) yield and nitrogen use efficiency by promoting symbiotic nitrogen fixation capacity

**DOI:** 10.3389/fpls.2025.1604251

**Published:** 2025-07-02

**Authors:** Yaxin Xu, Quantong Gao, Lihua Xue, Jianxin Zhang, Cong Wang

**Affiliations:** ^1^ College of Agriculture, Xinjiang Agricultural University, Urumqi, China; ^2^ Research Institute of Food Crops, Xinjiang Academy of Agricultural Sciences, Urumqi, China

**Keywords:** soybean, nitrogen fertilizer, symbiotic nitrogen fixation, nodulation, high yield

## Abstract

**Introduction:**

Although the mulched drip irrigation system combined with high nitrogen input (240∼310 kg ha^-1^) in Xinjiang, China, frequently achieves record-high soybean yields (6855 kg ha^-1^), this practice is not conducive to symbiotic nitrogen fixation and compromises agricultural sustainability.

**Methods:**

Under the mulched drip irrigation, this study evaluation four nitrogen application treatments (N_0_: 0 kg ha^-1^, N_120_: 120 kg ha^-1^, N_180_: 180 kg ha^-1^, and N_240_: 240 kg ha^-1^) were evaluated over two consecutive growing seasons to investigate their effects on nodule morphological and physiological traits, stem ureide content, and the percentage of nitrogen derived from the atmosphere (%Ndfa) during the reproductive growth stage.

**Results:**

The application of 180 kg ha^-1^ nitrogen significantly increased nodule number, nodule dry weight, nodule sucrose content, and nodule starch content, while improving soybean yield and nitrogen agronomic use efficiency. Conversely, the application of nitrogen exceeding 180 kg ha^-1^ inhibited nitrogenase activity, suppressed leghemoglobin synthesis, disrupted the glutamine synthetase/glutamate synthase metabolic pathway, and reduced ureide translocation from nodules to stems, leading to significant accumulation of ureides in nodules. Correlation and path analyses indicated that nitrogenase activity, leghemoglobin content, urate oxidase activity, and stem ureide content were significantly positively correlated with %Ndfa, whereas nodule ureide content showed a significant negative correlation with %Ndfa. Stem ureide content exhibited a strong direct positive effect on %Ndfa (path coefficient = 0.95), confirming its validity as a robust indicator for assessing SNF capacity.

**Discussion:**

In conclusion, mulched drip irrigation, applying 180 kg ha^-1^ nitrogen at the beginning pod stage (R_3_) effectively enhances root nodulation, promotes carbohydrate allocation to nodules, sustains symbiotic nitrogen fixation activity, and ultimately increases soybean yield and nitrogen use efficiency. Thus, under mulched drip irrigation system, applying the correct rate of nitrogen fertilizer is beneficial for enhancing soybean yield and mitigating environmental risks, which holds significant importance for promoting sustainable agricultural development.

## Introduction

Crop yields heavily depend on nitrogen (N) fertilizer supply. Owing to the low cost and high N content of urea, farmers often apply N fertilizers excessively and indiscriminately in pursuit of higher yields, leading to yield stagnation, significant declines in nitrogen use efficiency (NUE), and exacerbated environmental pollution through energy-intensive production and application (Liu et al., 2021; Bodirsky et al., 2014; Camargo et al., 2005). Consequently, enhancing NUE is of crucial importance for sustainable agriculture. In Xinjiang, under mulched drip irrigation combined with integrated water-fertilizer management has repeatedly achieved record-high soybean yields in China. However, the total N supply during the growing season remains excessively high (240∼310 kg ha^-1^), resulting in high yields but low NUE. This inefficiency stems from farmers adopting cotton field management practices for soybeans, ignoring the unique role of symbiotic nitrogen fixation (SNF) in soybean nitrogen acquisition, which differs fundamentally from graminaceous crops like cotton and rice. While soybean nitrogen sources include soil N, fertilizer N, and SNF, relying solely on SNF cannot achieve high yields; thus, targeted exogenous N supplementation based on soil fertility remains essential (Thorburn et al., 2024). Coordinating SNF, N fertilization, and yield by optimizing exogenous N supply to maximize SNF potential, establish an efficient soybean SNF system, and achieve high yield with resource efficiency holds significant practical importance (Goyal et al., 2021; Coba de la Peña et al., 2018).

Soybean plants acquire N through root uptake and SNF in root nodules. However, excessive N supply is well-documented to suppress root nodulation. When soil N availability increases significantly, plants prioritize direct root N absorption over SNF due to its lower energy cost, leading to reduced nodule number, nodule biomass, nitrogenase activity, leghemoglobin content, impaired glutamine synthetase (GS)/glutamate synthase (GOGAT) and glutamate dehydrogenase(GDH) assimilation pathways, and accelerated nodule senescence (Lyu et al., 2020; Albornoz, 2016; Wang et al., 2015). It is suggested by most studies that high N inputs inhibit root hair infection and nodule initiation (Mendes et al., 2018; Santachiara et al., 2018). Conversely, some researchers argue that low-level N fertilization does not compromise nodulation, as nodules may outcompete roots for resources; moderate N application during reproductive growth slightly reduces nodule weight but maintains nitrogenase activity (Ke et al., 2023; Pampana et al., 2018; Carter and Tegeder, 2016). Notably, Nguyen et al. (2020) demonstrated that novel rhizobial strains exhibit robust nodulation and SNF capacity even under high nitrate conditions. Proposed mechanisms for N-mediated suppression of SNF include carbon starvation (Lu et al., 2022; Parvin et al., 2020), nitrogen metabolite feedback regulation (Nazari et al., 2019; Castañeda et al., 2018), and internal oxygen limitation (Larrainzar et al., 2020; Gourion et al., 2015). Studies indicate that 50∼60% of soybean N demand is met via SNF (Salvagiotti et al., 2008), with some cases exceeding 90% (Mastrodomenico and Purcell, 2012). Strong positive correlations exist between nodule number, nodule dry weight, SNF efficiency, and seed yield, underscoring the critical role of nodule health in N fixation and productivity (Ferguson et al., 2019; Burias and Planchon, 1990). To optimize high-yield soybean N demand while minimizing exogenous N input, strategies such as deep placement of slow-release N fertilizers in nodulation zones (Yaseen et al., 2021), early sowing (Kurdali, 1996; Sprent and Thomas, 1984), and split N application after the beginning pod stage (R_3_) (Zapata et al., 1987) can sustain nodule activity and enhance yield efficiency.

Ureides (allantoin and allantoic acid), the primary SNF products exported from soybean root nodules to stems, are temporarily stored in stems (Atkins and Smith, 2007; Smith and Atkins, 2002). Enhancing ureide export from nodules positively impacts soybean reproductive growth by increasing SNF capacity and nodule number (Carter and Tegeder, 2016). In the enzymatic synthesis of ureides, urate oxidase (UO) activity catalyzes the oxidation of uric acid to allantoate, playing a critical role in nodule development and SNF. Ureide synthesis helps plants avoid ammonia toxicity by reducing glutamine and asparagine accumulation in nodules (Britto and Kronzucker, 2002; Engvild, 1987). Excessive N fertilization suppresses nodule uricase activity, elevates ammonia toxicity risks, and reduces stem ureide content (Wang et al., 2024). Under low N conditions, ureide export from nodules to stems is enhanced, whereas high N supply inhibits ureide translocation. Accumulated ureides in nodules directly suppress nitrogenase activity and indirectly impair SNF via feedback regulation mediated by stem ureide levels (Gil-Quintana et al., 2013; Serraj et al., 1999).

Previous studies have demonstrated that excessive exogenous N supply significantly suppresses soybean root nodulation and SNF (Thorburn et al., 2024). Under the mulched drip irrigation, this study systematically investigates the response mechanisms of root nodule developmental dynamics and nitrogen fixation metabolism in high-yield soybean to varying N levels. We evaluate the adaptability of soybean nodules under different N regimes from the perspectives of N fixation, assimilation, and translocation. We hypothesize that under mulched drip irrigation, excessive N application at the beginning of pod formation will impede nodule formation, downregulate nitrogenase activity, inhibit carbohydrate allocation to nodules, and reduce long-distance transport of ureides (allantoin and allantoic acid) to aboveground tissues.

## Materials and methods

### Experimental field and meteorological conditions

**Figure 1:**
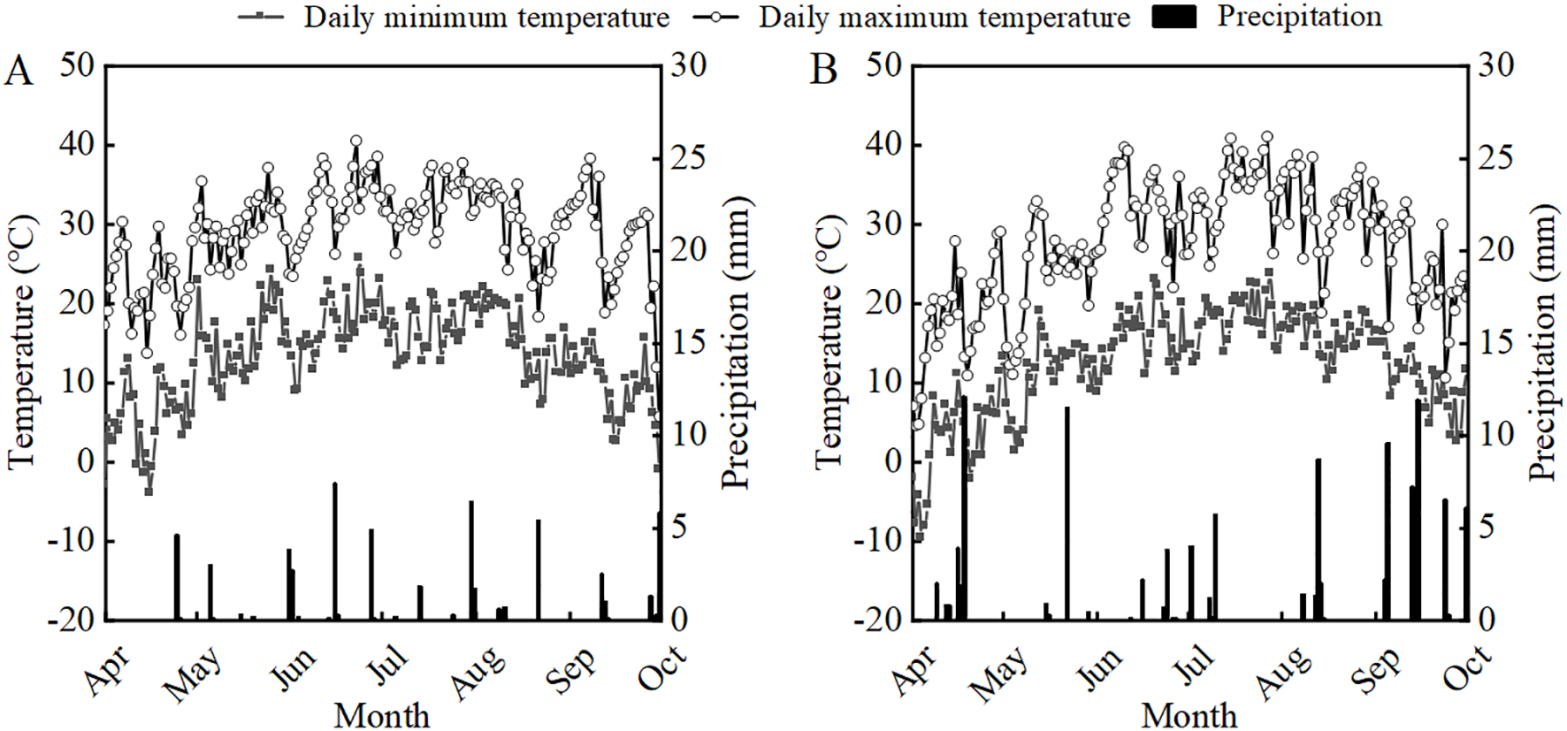
Daily minimum temperature, maximum temperature and rainfall from April to October in 2022 **(A)** and 2023 **(B)**.

The experiment was conducted from April 2022 to October 2023 at the Sanping Experimental Farm of Xinjiang Agricultural University in Urumqi, Xinjiang (43°56′N, 87°20′E). This region is characterized by a temperate continental arid climate. Daily maximum and minimum temperatures and precipitation during the soybean growing seasons are shown in **Figure 1**. The experimental field soil was sandy loam. Before sowing in 2022 and 2023, key soil nutrient parameters in the 0—20 cm layer were: organic matter (13.5 and 14.6 g kg^-1^), total nitrogen (0.91 and 0.73 g kg^-1^), alkali-hydrolyzable nitrogen (60.2 and 53.1 mg kg^-1^), available phosphorus (16.2 and 17.4 mg kg^-1^), and available potassium (185.2 and 216.0 mg kg^-1^), respectively.

### Experimental design and crop husbandry

The experiment followed a randomized complete block design. Four N treatments were implemented at the R_3_ stage: 0 (N_0_), 120 (N_120_), 180 (N_180_), and 240 kg ha^-^¹ (N_240_). Each plot measured 48 m² (4.8 m×10 m) with three replications, separated by 2 m buffer zones. Prior to tillage, calcium superphosphate (containing 19% P_2_O_5_) was applied at Soybeans were planted on April 27 and harvested from September 6 to 26 over two consecutive years. The planting used an equal row spacing of 40 cm, with a density 33×10^4^ plants ha^-1^. The field was mulched with 140 cm wide black plastic film, under which two labyrinth-type drip tapes (57 kg P_2_O_5_ ha^-1^. Φ16 mm with a discharge rate of 2.5–3.5 L h^-1^) were laid with 40 cm spacing between them. Irrigation was conducted every 10–13 days from June 17–27 to August 5–15 each year, with a total of 5 irrigations and a total water volume of 3300 m^3^ ha^-1^. Fertigation was implemented using monopotassium phosphate (containing 34% K_2_O and 52% P_2_O_5_), with total nutrient applications of 51 kg K_2_O ha^-1^ and 78 kg P_2_O_5_ ha^-1^, applied in a 1:2 ratio during the initial flowering and pod-setting stages. Two manual weeding were performed during the soybean growing season, with other management practices consistent with conventional field cultivation.

### Sample collection

At the full flowering stage (R_2_), full pod stage (R_4_), beginning pod stage (R_5_), full seed stage (R_6_), and physiological maturity stage (R_8_), 10 representative plants were selected from each treatment. Soybean roots were carefully excavated, washed with tap water, and nodules with diameter>1 mm were excised with a scalpel and counted. Five plants per treatment were separated into organs, dried to constant weight at 60°C in an oven, ground through a 1 mm sieve, and stored dry. Nodules from the remaining five plants were immediately stored at -80°C until analysis.

### Analytic determinations

#### Metabolite concentration

To determine sucrose, starch, ureides, and nitrate concentrations, dried nodule and stem samples were homogenized with 10 mL of distilled water and incubated in a 95°C water bath for 1 hour to obtain extracts. Supernatants were collected, and sucrose and starch contents in nodules were determined following the method of Xu et al (Xu et al., 2015). Ureide concentrations in nodules and stems were measured using the colorimetric assay described by Van Der Drift et al. (1970). Nitrate content in the main stem was analyzed via the salicylic acid method as outlined by Cataldo et al (Cataldo et al., 1975). Fresh nodules were used to quantify leghemoglobin content according to the protocol of Smagghe et al. (2009).

#### Enzymatic activities

Nitrogenase activity was determined using the acetylene reduction assay (David et al., 1980). Fresh nodules (1 g) were placed in 8 mL sealed reaction vials. After withdrawing 1 mL of air with a sterile syringe, an equal volume of acetylene was injected. The vials were incubated at 25°C for 30 min, and ethylene production was measured via gas chromatography. Fresh nodules were homogenized in an appropriate extraction buffer, and the supernatant was collected for enzymatic assays: glutamine synthetase (GS), glutamate synthase (GOGAT), glutamate dehydrogenase (GDH), and urate oxidase (UO). GS activity was assayed following the method of O’Neal and Joy (1974). The reaction mixture contained 50 mM Tris-HCl (pH 7.5), 4 mM ATP, 80 mM sodium glutamate, 30 mM MgSO_4_, 10 mM NH_2_OH, and 30 mM cysteine. A standard curve was generated using γ-glutamyl hydroxamate. GOGAT activity was measured according to Groat and Vance (1981). The reaction medium consisted of 100 mM potassium phosphate buffer (pH 7.6), 0.1% (v/v) 2-mercaptoethanol, 100 μM NADH, 2.5 mM 2-oxoglutarate, and 100 mM glutamine. GDH activity was determined using the method of Bulen (1956). The reaction system included 0.1 M Tris-HCl (pH 7.5), 0.33 M 2-oxoglutarate, 3 M NH_4_Cl, and 0.2 mL NADH. UO activity was analyzed following Pineda et al. (1984). The reaction mixture contained 0.1 mM uric acid and 100 mM Tris-HCl (pH 8.5).

### Estimation of relative symbiotic nitrogen fixation (%Ndfa)

The relative abundance of ureides (RAU), a parameter characterizing SNF, was estimated using the concentrations of ureides and nitrate in soybean main stems, following Equation 1 as described by Herridge and Peoples (1990).


(1) $$\text{RAU}=\frac{4\text{a}}{4\text{a}+\text{b}}\times 100\%$$

Where:

RAU: relative abundance of ureide (%).

a and b: concentrations of ureide and nitrate (μmol g^-1^), respectively.

Ureide contains four N atoms, NO_3_^-^contains one N atom.

The percentage of nitrogen derived from the atmosphere (%Ndfa) during soybean growth was estimated using the relative ureide content in main stems via a calibration equation (Herridge and Peoples, 1990). The calibration equation was established based on the ¹^5^N isotopic dilution technique and cross-validated against two independent methods: the ¹^5^N isotope dilution approach for multiple crops (Peoples et al., 1996) and the ureide-based %Ndfa estimation method (Alves et al., 2000). Both methods demonstrated a strong correlation. Ndfa was calculated using calibration Equation 2.


(2) $$\%\text{Ndfa}=-0.007\;4\;{\text{RAU}}^{2}+1.68\text{ RAU}+4.36$$

### Yield and NUE

At maturity stage (R_8_), 10 representative soybean plants were selected per treatment with 3 replicates, and the number of pods per plant, number of seeds per plant, and 100-seed weight were recorded. For yield determination, a 6.4 m^2^ (1.6 m×4 m) area was harvested with three replications. Harvested seeds were air-dried, weighed, and plot yield was converted to standard moisture content of 13%. NUE was calculated based on yield data as follows:


$$\text{NUE}=\frac{\text{Yield of nitrogen}-\text{treated plots – Yield of nitrogen}-\text{free plots}}{\text{Nitrogen application rate}}$$

### Data analysis

An analysis of variance (ANOVA) was employed to assess the effects of N treatments across different years and growth stages on enzyme activities, ureide content, sucrose, and starch. A two-way ANOVA was conducted to evaluate the interactive effects of year and N application rate on nodule number, nodule dry weight, nitrogenase activity, leghemoglobin content, and yield. Data are presented as mean. *Post hoc* comparisons were carried out with LSD. Duncan’s multiple range test and Pearson correlation analyses were implemented in SPSS 26.0 (SPSS Inc., Chicago, IL, USA). Structural equation modeling (SEM) was fitted via the graphical interface of IBM SPSS AMOS 26 to quantify the influence of SNF indicators on %Ndfa.

## Results

### Nodules parameters

The analysis of variance (**Table 1**) revealed that, from the R_4_ to R_6_ stages, both year and N application rate significantly affected nodule number and nodule dry weight (The significance level for the following data was set at *p*<0.05), and their interaction significantly influenced nodule dry weight. Across the 2022 and 2023 growing seasons, nodule number and dry weight initially increased and then decreased with advancing growth stages, peaking at the R_5_ stage before declining. The N_240_ treatment exhibited the most pronounced reduction, with decreases of 41.23% and 37.25% in nodule number and dry weight, respectively. From R_4_ to R_6_, nodule number and dry weight displayed a unimodal response to increasing N rates, reaching maximum values under the N_180_ treatment. Compared to N_180_, the N_240_ treatment reduced nodule number by 72.57%, 65.66%, and 56.14% at R_4_, R_5_, and R_6_ stages, respectively, and decreased nodule dry weight by 58.22%, 67.46%, and 67.05%.

**Table 1:** Effect of nitrogen application on nodule number and dry weight of soybean.

Year	Treatment	Nodules (nodules m^-2^)				Nodule dry weight (g m^-2^)			
(Y)	(T)	R_2_	R_4_	R_5_	R_6_	R_2_	R_4_	R_5_	R_6_
2022	N_0_	305.25a	742.50c	1311.75c	1039.5a	3.70a	4.02c	12.52c	8.40b
	N_120_	330.00a	1064.25b	1963.50b	1097.25a	3.89a	8.49b	18.77b	10.24a
	N_180_	305.25a	1600.50a	2433.75a	1427.25a	3.80a	11.00a	20.06a	12.17a
	N_240_	305.25a	412.50d	701.25d	478.50b	3.67a	2.78c	3.90d	3.11c
2023	N_0_	239.25a	1064.25b	1369.50c	1278.75b	2.46a	16.67a	19.42b	18.13b
	N_120_	264.00a	1212.75b	2367.75b	1460.25b	2.44a	17.24a	22.89a	16.24b
	N_180_	272.25a	1765.50a	2813.25a	1765.50a	2.47a	15.44a	25.44a	21.06a
	N_240_	255.75a	511.50c	1122.00d	957.00c	2.52a	9.00b	11.61c	8.50c
Y		**	**	**	**	ns	**	**	**
T		ns	**	**	**	ns	**	**	**
Y×T		ns	ns	ns	ns	ns	*	*	*

Data are expressed as means (*n*=3). Different letters indicate a statistically significant level at *p*<0.05. ns, * and ** indicate nonsignificant, significant at 5% and 1% level, respectively.

### Nodule nitrogenase activity and leghemoglobin

The ANOVA results (**Table 2**) indicated that, from the R_4_ to R_6_ stages, year, N application rate, and their interaction significantly influenced nodule nitrogenase activity and leghemoglobin content. Across the 2022 and 2023 growing seasons, nitrogenase activity and leghemoglobin content in all treatments initially increased and then decreased with advancing growth stages, peaking at the R_5_ stage. At the R_4_ and R_5_ stages, nitrogenase activity and leghemoglobin content gradually decreased with increasing N application. Compared to other treatments, the N_240_ treatment reduced nitrogenase activity by 63.37%∼78.91% and 56.94%∼9.96%, respectively, and leghemoglobin content by 35.82%∼61.21% and 44.74%∼63.69%. At the R6 stage, nitrogenase activity and leghemoglobin content in the N_240_ treatment decreased by 66.72%∼376.54% and 9.57%∼140.40%, respectively.

**Table 2:** Effect of nitrogen application on nodule nitrogenase activity and leghemoglobin.

Year(Y)	Treatment(T)	Nodule nitrogenase activity (μmol g^-1^ h^-1^)				Leghemoglobin (mg g^-1^)			
		R_2_	R_4_	R_5_	R_6_	R_2_	R_4_	R_5_	R_6_
2022	N_0_	3.54a	8.44a	9.43a	6.11b	3.6a	8.32a	11.78a	7.47a
	N_120_	3.24a	6.56b	7.99b	7.72a	3.44a	7.34a	10.37b	6.01b
	N_180_	3.37a	4.86c	6.54c	4.88c	3.26a	5.22b	6.56c	3.14c
	N_240_	3.34a	1.78d	1.89d	1.62d	3.19a	2.89c	3.94d	2.50c
2023	N_0_	4.16a	9.50a	6.94a	5.89a	4.86a	9.15a	15.03a	6.44a
	N_120_	3.77a	7.16b	6.63a	5.33a	4.35a	7.30b	12.38b	7.40a
	N_180_	3.69a	6.75b	4.97b	4.35b	4.35a	5.37c	8.45c	4.81b
	N_240_	3.61a	2.43c	2.14c	1.96c	4.34a	3.92d	4.29d	4.39b
Y		**	**	**	*	**	*	**	**
T		ns	**	**	**	ns	**	**	**
Y×T		ns	*	**	ns	ns	*	**	**

Data are expressed as means (*n*=3). Different letters indicate a statistically significant level at *p*<0.05. ns, * and ** indicate nonsignificant, significant at 5% and 1% level, respectively.

### Enzymes involved in nitrogen metabolism of nodules

**Figure 2:**
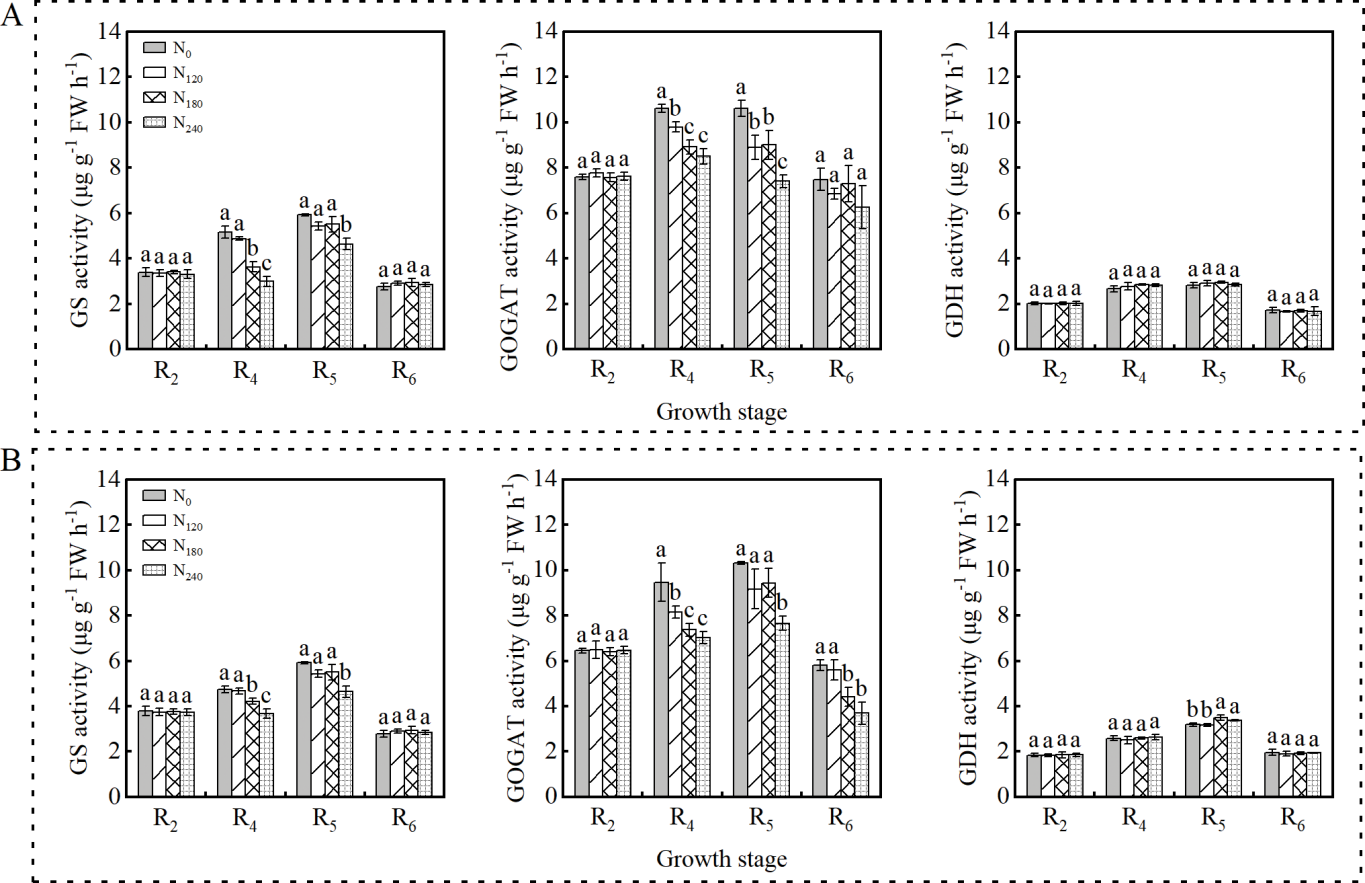
Effects of nitrogen application on nitrogen metabolism enzyme activities in nodules in 2022 **(A)** and 2023 **(B)**. Bars represent means and error bars standard error (*n*=3). Different letters represent significant differences (*p*<0.05) between treatments at the same growth stage.

As shown in **Figure 2**, GS, GOGAT, and GDH activities in nodules exhibited a unimodal trend during both the 2022 and 2023 growing seasons, peaking at the R_5_ stage before declining. Increased N application at the R_4_ and R_5_ stages significantly influenced GS and GOGAT activities, whereas GDH activity remained unaffected. Similar trends were observed between the two growing seasons.

### Urate oxidase activity of nodule

**Figure 3:**
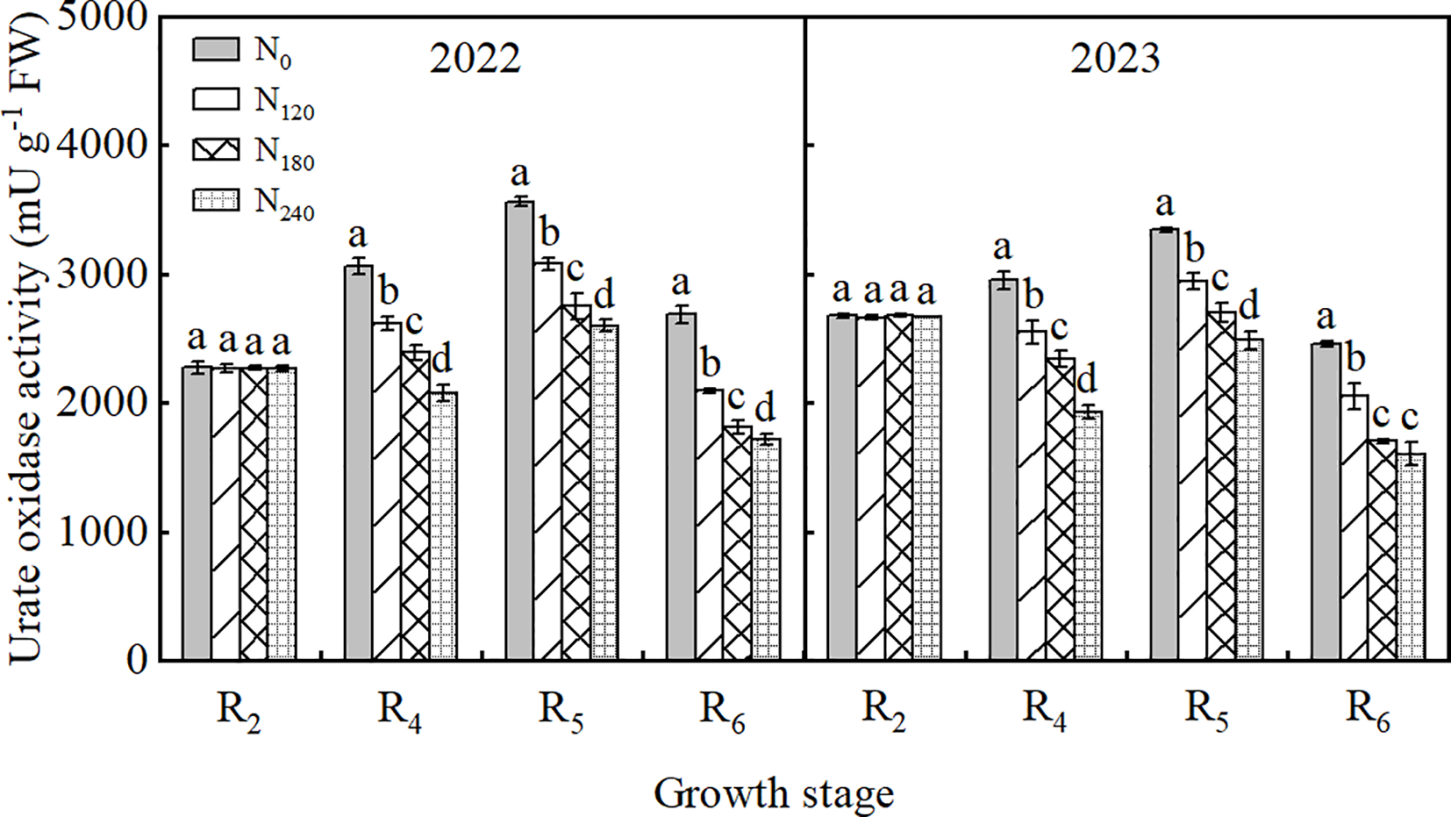
Effect of nitrogen application on the activities of urate oxidase in soybean nodules in 2022 and 2023. Bars represent means and error bars standard error (*n*=3). Different letters represent significant differences (*p*<0.05) between treatments at the same growth stage.

As shown in **Figure 3**, during both 2022 and 2023 growing seasons, UO activity in nodules first increased and then decreased with advancing growth stages, peaking at the R_5_ stage. UO activity in the N_240_ treatment was significantly reduced by 5.68%∼27.14% and 8.01%∼25.68% compared to other treatments in 2022 and 2023, respectively. Increasing N supply significantly decreased UO activity, following the order N_0_>N_120_>N_180_>N_240_.

### Ureide content

**Figure 4:**
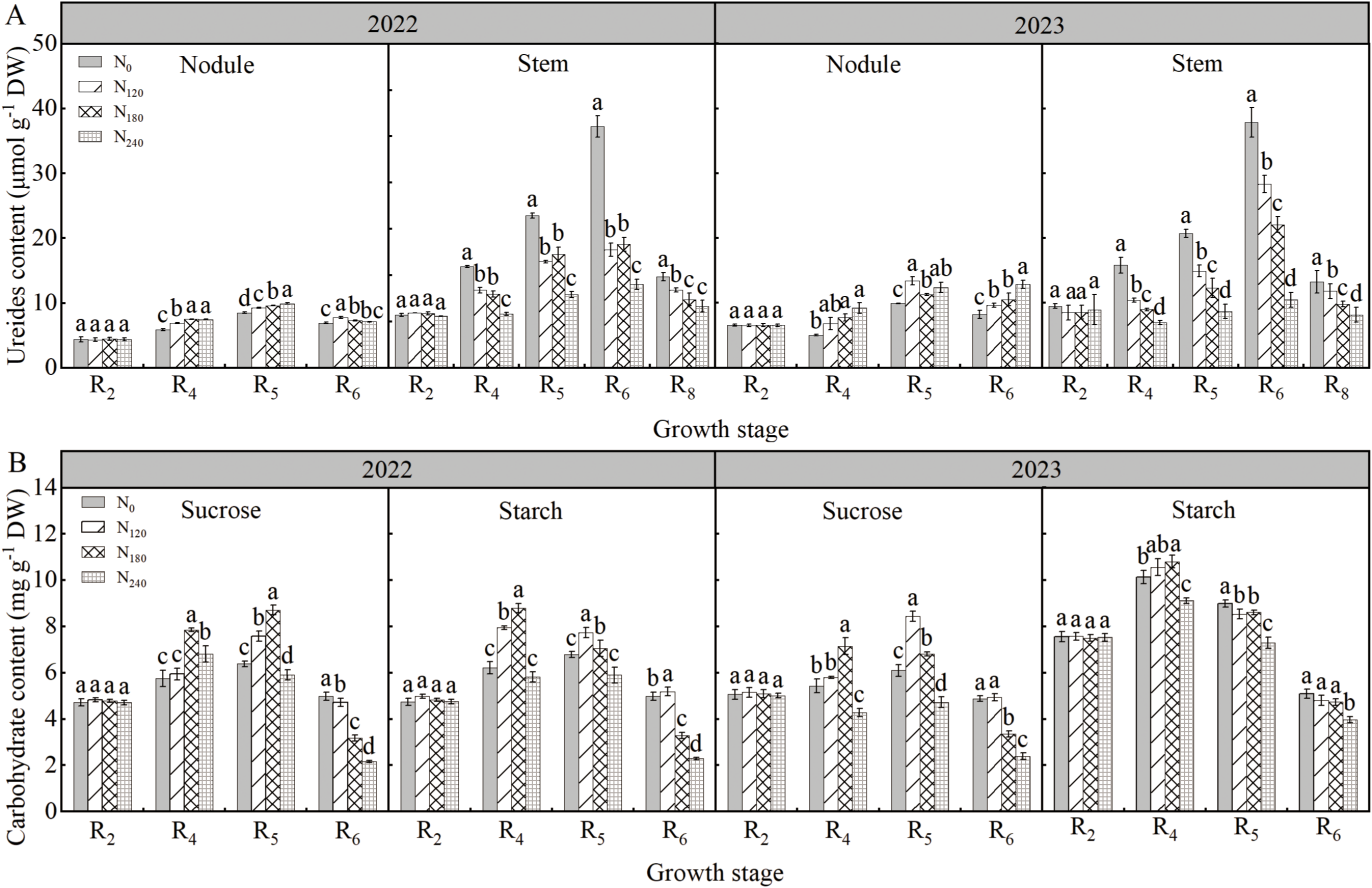
Effects of nitrogen application on ureide content **(A)** and nodule carbohydrate content **(B)** in 2022 and 2023. Bars represent means and error bars standard error (*n*=3). Different letters represent significant differences (*p*<0.05) between treatments at the same growth stage.

As shown in **Figure 4A**, during both 2022 and 2023 growing seasons, ureide contents in nodules and stems first increased and then decreased with advancing growth stages, peaking at R_5_ and R_6_ stages, respectively. Nodule ureide content in the N_240_ treatment was significantly higher by 3.00%∼24.77% compared to other treatments, while stem ureide content was reduced by 29.15%∼72.31%. Increasing N application promoted ureide accumulation in nodules but showed the opposite trend in stems.

### Sucrose and starch content of nodules

As shown in **Figure 4B**, during both 2022 and 2023 growing seasons, sucrose and starch contents in nodules first increased and then decreased with advancing growth stages, peaking at the R_5_ and R_4_ stages, respectively. At the R_4_ stage across both years, nodule sucrose content was highest under the N_180_ treatment, increasing by 15.26%∼36.30% and 23.17%∼31.62% compared to other N treatments, respectively. Nodule starch content under N_180_ increased by 10.59%∼41.39% and 2.30%∼18.59% compared to other N treatments in the two years.

### Grain yield and NUE

ANOVA results (**Table 3**) indicated that year and N application rate significantly affected pod number per plant, seed number per plant, yield, and %Ndfa. In 2022, N treatments increased pod number per plant by 20.40%∼34.46%, seed number per plant by 10.34%∼22.86%, and 100-seed weight by 6.90%∼13.80% compared to N_0_, with consistent trends observed in 2023. The N_180_ achieved the highest yield across both years, increasing by 6.20%∼19.23% and 12.61%∼24.92% compared to other treatments. N_180_ significantly improved NUE, with NUE increases of 5.96% and 57.14% over N_120_ and N_240_ in 2022, and 31.26% and 175% in 2023. %Ndfa gradually decreased with increasing N application, with reductions of 14.07%∼33.16% and 10.83%∼37.87% in 2022 and 2023, respectively, compared to N_0_.

**Table 3:** Effects of nitrogen application on grain yield and yield components.

Year	Treatment	Pods per plant	Seeds per plant	100-grain weight	Yield	NUE	Ndfa
(Y)	(T)			(g)	(kg ha^-1^)	(kg kg^-1^)	(%)
2022	N_0_	25.25d	77.85d	18.40c	3433.36c		37.95a
	N_120_	30.40c	85.90c	19.67b	3721.68b	6.38a	32.61b
	N_180_	37.60a	95.65a	19.87b	4096.68a	6.76a	28.92c
	N_240_	33.95b	89.45b	20.94a	3857.54b	5.16b	25.37d
2023	N_0_	28.40c	79.70c	18.44c	3525.94c		42.28a
	N_120_	32.95b	88.95b	19.26b	3881.50b	6.16b	37.70b
	N_180_	36.90a	98.85a	19.87ab	4404.47a	9.68a	32.33c
	N_240_	34.00b	90.05b	20.29a	3911.13b	3.52c	26.27d
Y		*	*	ns	**	ns	**
T		**	**	**	**	**	**
Y×T		ns	ns	ns	ns	**	ns

Data are expressed as means (*n*=3). Different letters indicate a statistically significant level at *p*<0.05. ns, * and ** indicate nonsignificant, significant at 5% and 1% level, respectively.

### The relationship between the parameters of SNF in soybean and %Ndfa

**Figure 5:**
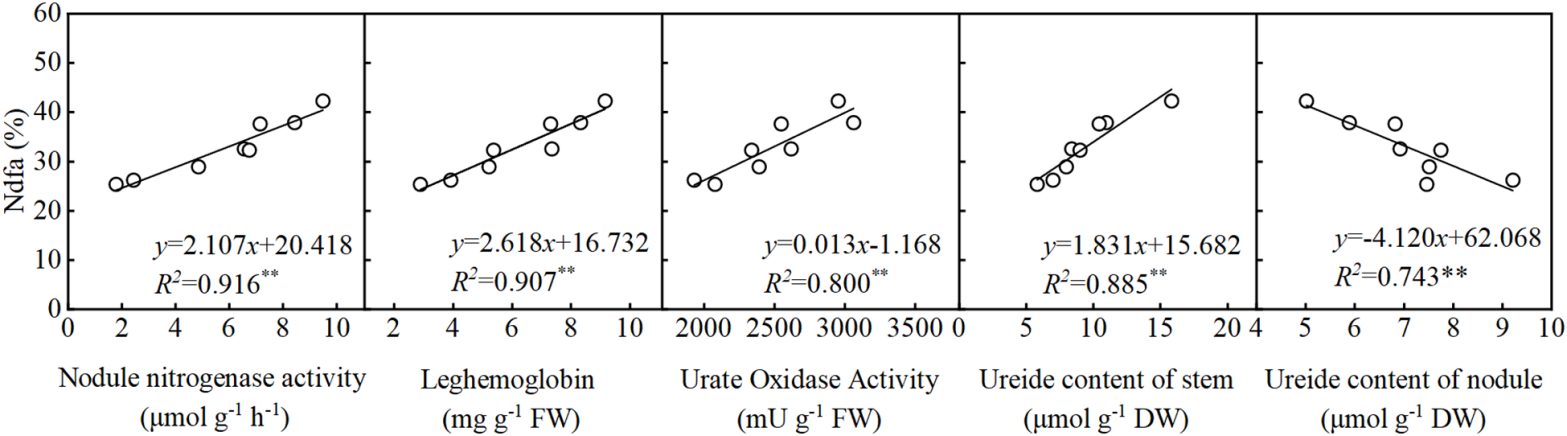
The relationship between the parameters of symbiotic nitrogen fixation in soybean and %Ndfa at the full pod stage (R_4_).

**Figure 6:**
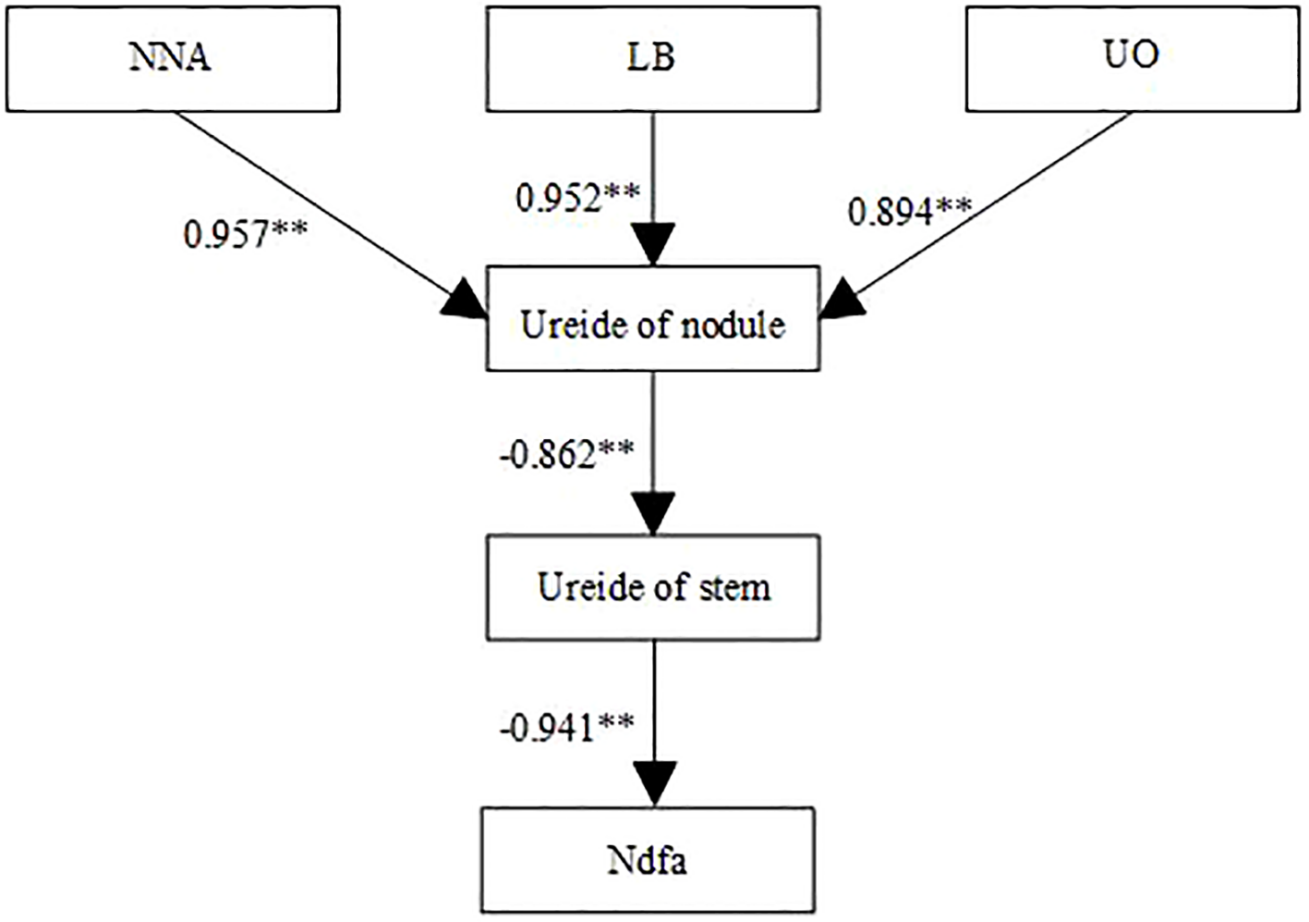
Structural equation modeling (SEM) of the relationships between symbiotic nitrogen fixation traits and nitrogen derived from atmosphere (%Ndfa) at R_4_ stage in 2022 and 2023. Solid and dashed arrows indicate significant and nonsignificant path coefficients, respectively. NNA, Nodule nitrogenase activity, LB, Leghemoglobin content, UO: Urate oxidase activity. Values above arrows represent standardized path coefficients. ** denote significance at the 0.01 probability levels.

As shown in **Figure 5**, nodule nitrogenase activity, leghemoglobin content, UO activity, and stem ureide content all exhibited significant positive correlations with %Ndfa. Conversely, nodule ureide content showed a significant negative correlation with %Ndfa. Structural equation modeling (SEM) was used to evaluate the effects of SNF indicators on %Ndfa. UO activity significantly negatively influenced nodule ureide content, with a path coefficient of -0.93 (**Figure 6**). Nodule ureide content significantly negatively affected stem ureide content. Stem ureide content exerted a highly significant positive effect on %Ndfa, with a path coefficient of 0.95.

## Discussions

In well-managed soybean fields, SNF can supply 70∼85% of the crop’s N demand (Alves et al., 2003). Accumulating evidence from numerous studies indicates that high levels of N in the soil strongly inhibit rhizobial infection efficiency, nodule organogenesis, and nitrogen fixation efficiency, manifesting as reduced nodule number, decreased nodule biomass, inhibited nitrogenase activity, and accelerated nodule senescence or degradation (Pampana et al., 2018; Carter and Tegeder, 2016; Albornoz, 2016; Wang et al., 2015). Conversely, low levels of N enhance nodulation and nitrogen fixation activity (Ke et al., 2023). Given evidence that R_3_ stage N application significantly impacts grain yield (Mourtzinis et al., 2018; Zapata et al., 1987), this study applied N treatments at R_3_ stage, resulting in no significant differences in nodule morpho-physiological traits across treatments at the R_2_ stage. In our experiment, the application of 180 kg ha^-1^ nitrogen increased nodule biomass and number, while the application of N at rates exceeding 180 kg ha^-1^ caused significant declines in both parameters. These findings partially contrast with those of Gan et al. (2002) and Nanjareddy et al. (2014), likely due to the lower initial soil N levels in our experimental plots, where N was not the primary limiting factor for nodulation. While nodulation is foundational to SNF, high nodule numbers do not necessarily correlate with high nitrogen fixation capacity (Herridge and Rose, 2000; Song et al., 1995). Extensive research demonstrates that nitrogenase activity strongly links to SNF efficiency and yield, with elevated soil mineral suppressing nitrogenase activity and leghemoglobin content (Liu et al., 2022; Lyu et al., 2020). Our results corroborate this: higher soil N concentrations reduced nodule nitrogenase activity, likely due to diminished leghemoglobin content, which compromises oxygen regulation in infected nodule cells. Since nitrogenase is highly oxygen-sensitive, this dysregulation leads to marked declines in enzymatic activity (Larrainzar et al., 2020; Kawashima et al., 2001). Consistently, our data confirm that N fertilization reduces nodule leghemoglobin content.

Nitrogen availability significantly impacts ammonium assimilation in soybean nodules: elevated soil N enhances GS and GOGAT activities in roots but suppresses these enzymes in nodules, particularly GOGAT (Sawhney et al., 1985). This study also reached the same conclusion, increased N application reduced both GS and GOGAT activities, with GOGAT showing greater sensitivity, consistent with Do Amarante et al. (2022).To prevent excessive ammonium accumulation from inhibiting plant growth, the GDH pathway is activated to promote ammonium assimilation. In this study, nodule GDH activity increased with higher N application. Although GDH activity did not differ significantly among treatments in 2022, these trends still highlight the critical role of GDH in reducing NH_3_ toxicity in nodules, suggesting a potential shift in ammonium assimilation from the GS/GOGAT pathway to the GDH pathway. While high soil N concentrations transiently affect the nodule GDH pathway, this effect is not persistent. As plants absorb soil N, the inhibitory effect on the GDH pathway is alleviated, likely due to nonsignificant differences in GDH activity among treatments at R_6_ stage. This phenomenon may also be attributed to accelerated root and nodule senescence under low N conditions during the late growth stages (Nishida and Suzaki, 2018).

Besides ammonium assimilation, the synthesis and transport of ureide compounds in nodules also play crucial roles in the nitrogen metabolism of soybean. High N application elevates ureide content in nodules (Yamashita et al., 2019). In this study, under N-free conditions, nodule ureide export to stems and their allocation were significantly increased compared to other treatments, supported by reduced nodule ureide content and elevated stem ureide content. When N was applied, we observed significant reductions in nodule ureide synthase activities (e.g., UO), yet nodule ureide content remained higher than in N-free treatments, with the opposite trend in stems. This indicates that N application impairs ureide synthesis and transport in nodules, leading to ureide accumulation. Accumulated ureides likely exert feedback inhibition on nitrogenase activity, as evidenced by significantly reduced nitrogenase activity under high N conditions, consistent with findings by Serraj et al. (1999) and Gil-Quintana et al. (2013). Nodules act as strong carbon sinks, requiring energy for ureide synthesis. Thus, insufficient carbon transport to nodules limits SNF and related processes (Lu et al., 2022; Parvin et al., 2020). As the primary form of carbon transport, sucrose accumulation in nodules is closely linked to nitrogen fixation and assimilation. In this study, nodule sucrose and starch contents were lower in N-free and high-N treatments compared to other treatments, particularly in high-N plots. Reduced nodule carbon availability may be associated with decreased ureide export (Rawsthorne et al., 1980), resulting in elevated nodule ureide content, suppressed nitrogen fixation, accelerated nodule senescence, and reduced nitrogen use efficiency.

Although low N levels restrict soybean growth, high N application only marginally enhances yield, while excessive N supply reduces both yield and NUE (Thorburn et al., 2024).In this study, soybean yield reached its maximum at a N application rate of 180 kg ha^-1^, primarily attributed to increased pod and seed numbers. During the reproductive growth stage, reliance solely on SNF cannot fully meet plant N demand, necessitating supplemental N fertilization. Moderate N application compensates for N deficits, balances vegetative and reproductive growth, and provides energy for pod formation and grain filling, while simultaneously maximizing NUE. To evaluate relationships among nodule growth, stem ureide content, and %Ndfa at the R_4_ stage, correlation and structural equation modeling revealed significant positive correlations between nitrogenase activity, leghemoglobin content, UO activity, stem ureide content, and %Ndfa, consistent with van Berkum et al. (1985). Conversely, nodule ureide content showed a significant negative correlation with %Ndfa, further confirming that high N inhibits ureide translocation from nodules. Nitrogenase activity and leghemoglobin content positively regulated nodule ureide levels, whereas UO activity exhibited negative regulation, suggesting feedback control of UO activity by ureide accumulation. Nodule ureide content inversely influenced stem ureide content, while stem ureide content strongly and positively affected %Ndfa (path coefficient = 0.95). This explains why stem ureide content serves as a reliable proxy for nitrogenase activity, given that ureides account for approximately 80% of total stem N, aligning with findings by Mcclure and Israel (1979).

## Conclusion

Achieving simultaneous improvements in NUE and yield stability in future soybean production represents a core challenge for sustainable agriculture. Across the 2022 and 2023 growing seasons, our data demonstrate that under mulched drip irrigation, the application of 180 kg ha^-1^ nitrogen significantly enhanced root nodulation capacity (95.5% increase in nodule number and 45.6% increase in nodule dry weight at the R_5_ stage) and optimized nodule nitrogenase activity (5.8 µmol g^-1^ h^-1^ at the R_5_ stage) and leghemoglobin synthesis (7.5 mg g^-1^ at the R_5_ stage). These improvements elevated NUE to 8.2 kg kg^-1^ and increased yield by 22.1%. However, the application of N exceeding 180 kg ha^-1^ suppressed SNF (35.5% reduction in SNF) by impairing root nodulation capacity, inhibiting the GS/GOGAT metabolic pathway, and reducing ureide compound translocation from nodules to stems, leading to significant ureide accumulation in nodules. SEM revealed the stem ureide content exhibited a strong direct positive effect on %Ndfa (path coefficient=0.95), establishing stem ureide content as an effective physiological indicator for evaluating SNF capacity. Given the strong dependence of soybean nodule nitrogen fixation capacity on climatic conditions and agronomic practices, future research should focus on understanding interactions between SNF and environmental conditions to optimize adaptability, thereby achieving synergistic improvements in yield and NUE for future cultivars.

## Data Availability

The original contributions presented in the study are included in the article/**Supplementary Material**. Further inquiries can be directed to the corresponding authors.
