# Supplementary material for: Optimized nitrogen fertilizer management enhances soybean (Glycine max (L.) Merril.) yield and nitrogen use efficiency by promoting symbiotic nitrogen fixation capacity
Source: Front Plant Sci. 2025 Jul 2;16:1604251. doi: 10.3389/fpls.2025.1604251 (PMC12263379; doi:10.3389/fpls.2025.1604251)
Supplement: Supplementary file 1 [file Table1.docx]

**TABLE 1**

| Year | Treatment | Nodules (nodules m^-2^) | | | | Nodule dry weight (g m^-2^) | | | |
| --- | --- | --- | --- | --- | --- | --- | --- | --- | --- |
| (Y) | (T) | R_2_ | R_4_ | R_5_ | R_6_ | R_2_ | R_4_ | R_5_ | R_6_ |
| 2022 | N_0_ | 305.25a | 742.50c | 1311.75c | 1039.5a | 3.70a | 4.02c | 12.52c | 8.40b |
|  | N_120_ | 330.00a | 1064.25b | 1963.50b | 1097.25a | 3.89a | 8.49b | 18.77b | 10.24a |
|  | N_180_ | 305.25a | 1600.50a | 2433.75a | 1427.25a | 3.80a | 11.00a | 20.06a | 12.17a |
|  | N_240_ | 305.25a | 412.50d | 701.25d | 478.50b | 3.67a | 2.78c | 3.90d | 3.11c |
| 2023 | N_0_ | 239.25a | 1064.25b | 1369.50c | 1278.75b | 2.46a | 16.67a | 19.42b | 18.13b |
|  | N_120_ | 264.00a | 1212.75b | 2367.75b | 1460.25b | 2.44a | 17.24a | 22.89a | 16.24b |
|  | N_180_ | 272.25a | 1765.50a | 2813.25a | 1765.50a | 2.47a | 15.44a | 25.44a | 21.06a |
|  | N_240_ | 255.75a | 511.50c | 1122.00d | 957.00c | 2.52a | 9.00b | 11.61c | 8.50c |
| Y | | ** | ** | ** | ** | ns | ** | ** | ** |
| T | | ns | ** | ** | ** | ns | ** | ** | ** |
| Y×T | | ns | ns | ns | ns | ns | * | * | * |

**TABLE 2**

| Year(Y) | Treatment(T) | Nodule nitrogenase activity  (μmol g^-1^ h^-1^) | | | | Leghemoglobin (mg g^-1^) | | | |
| --- | --- | --- | --- | --- | --- | --- | --- | --- | --- |
|  |  | R_2_ | R_4_ | R_5_ | R_6_ | R_2_ | R_4_ | R_5_ | R_6_ |
| 2022 | N_0_ | 3.54a | 8.44a | 9.43a | 6.11b | 3.6a | 8.32a | 11.78a | 7.47a |
|  | N_120_ | 3.24a | 6.56b | 7.99b | 7.72a | 3.44a | 7.34a | 10.37b | 6.01b |
|  | N_180_ | 3.37a | 4.86c | 6.54c | 4.88c | 3.26a | 5.22b | 6.56c | 3.14c |
|  | N_240_ | 3.34a | 1.78d | 1.89d | 1.62d | 3.19a | 2.89c | 3.94d | 2.50c |
| 2023 | N_0_ | 4.16a | 9.50a | 6.94a | 5.89a | 4.86a | 9.15a | 15.03a | 6.44a |
|  | N_120_ | 3.77a | 7.16b | 6.63a | 5.33a | 4.35a | 7.30b | 12.38b | 7.40a |
|  | N_180_ | 3.69a | 6.75b | 4.97b | 4.35b | 4.35a | 5.37c | 8.45c | 4.81b |
|  | N_240_ | 3.61a | 2.43c | 2.14c | 1.96c | 4.34a | 3.92d | 4.29d | 4.39b |
| Y | | ** | ** | ** | * | ** | * | ** | ** |
| T | | ns | ** | ** | ** | ns | ** | ** | ** |
| Y×T | | ns | * | ** | ns | ns | * | ** | ** |

**TABLE 3**

| Year | Treatment | Pods per plant | Seeds per plant | 100-grain weight | Yield | NUE | Ndfa |
| --- | --- | --- | --- | --- | --- | --- | --- |
| (Y) | (T) |  |  | (g) | (kg ha^-1^) | (kg kg^-1^) | (%) |
| 2022 | N_0_ | 25.25d | 77.85d | 18.40c | 3433.36c |  | 37.95a |
|  | N_120_ | 30.40c | 85.90c | 19.67b | 3721.68b | 6.38a | 32.61b |
|  | N_180_ | 37.60a | 95.65a | 19.87b | 4096.68a | 6.76a | 28.92c |
|  | N_240_ | 33.95b | 89.45b | 20.94a | 3857.54b | 5.16b | 25.37d |
| 2023 | N_0_ | 28.40c | 79.70c | 18.44c | 3525.94c |  | 42.28a |
|  | N_120_ | 32.95b | 88.95b | 19.26b | 3881.50b | 6.16b | 37.70b |
|  | N_180_ | 36.90a | 98.85a | 19.87ab | 4404.47a | 9.68a | 32.33c |
|  | N_240_ | 34.00b | 90.05b | 20.29a | 3911.13b | 3.52c | 26.27d |
| Y | | * | * | ns | ** | ns | ** |
| T | | ** | ** | ** | ** | ** | ** |
| Y×T | | ns | ns | ns | ns | ** | ns |
